# Analysis of the dominant mutation N188T of human connexin46 (hCx46) using concatenation and molecular dynamics simulation

**DOI:** 10.1002/2211-5463.12624

**Published:** 2019-03-23

**Authors:** Patrik Schadzek, Yannick Stahl, Matthias Preller, Anaclet Ngezahayo

**Affiliations:** ^1^ Institute of Cell Biology and Biophysics Department of Cell Physiology and Biophysics Leibniz University Hannover Germany; ^2^ Institute for Biophysical Chemistry Hannover Medical School (MHH) Germany; ^3^ Centre for Structural Systems Biology, DESY‐Campus Hamburg Germany; ^4^ Center for System Neurosciences (ZSN) Hannover Germany

**Keywords:** cataract, concatenation, dominant inheritance, hCx46, hCx46N188T, molecular dynamics

## Abstract

Connexins (Cx) are proteins that form cell‐to‐cell gap junction channels. A mutation at position 188 in the second extracellular loop (E2) domain of hCx46 has been linked to an autosomal dominant zonular pulverulent cataract. As it is dominantly inherited, it is possible that the mutant variant affects the co‐expressed wild‐type Cx and/or its interaction with other cellular components. Here, we proposed to use concatenated hCx46wt‐hCx46N188T and hCx46N188T‐hCx46wt to analyze how hCx46N188T affected co‐expressed hCx46wt to achieve a dominant inheritance. Heterodimer hCx46wt‐hCx46N188T formed fewer gap junction plaques compared to homodimer hCx46wt‐hCx46wt, while the hCx46N188T‐hCx46N188T homodimer formed almost no gap junction plaques. Dye uptake experiments showed that hemichannels of concatenated variants were similar to hemichannels of monomers. Molecular dynamics simulations revealed that for docking, the N188 of a protomer was engaged in hydrogen bonds (HBs) with R180, N189, and D191 of the counterpart protomer of the adjacent hemichannel. T188 suppressed the formation of HBs between protomers. Molecular dynamics simulations of an equimolar hCx46wt/hCx46N188T gap junction channel revealed a reduced number of HBs between protomers, suggesting reduction of gap junction channels between lens fibers co‐expressing the variants.

**Abbreviations**
Cx: connexin
E1: first extracellular loop
E2: second extracellular loop
Edt: ethidium bromide
HB: hydrogen bond
hCx: human connexin
LY: Lucifer yellow
MD: molecular dynamics
ns: not significant
wt: wild‐type

Connexins (Cx) are proteins that form cell‐to‐cell gap junction channels. The gap junction channels allow exchange of ions and small metabolites between cells in a tissue leading, thereby to formation of synchronized physiological units within a tissue 1. To form a gap junction channel, hemichannels within the membrane of adjacent cells dock to each other generating a cell‐to‐cell pore that allows diffusion of small metabolites up to 1–2 kDa between the cytoplasmic space of the interacting cells. Upon docking the gap junction, channels are assembled in gap junction plaques, which might contain more than thousand channels 2. A hemichannel is composed of six Cx subunits that oligomerize along the traffic pathway between the endoplasmic reticulum and the trans‐Golgi network 3, 4, 5, 6. They are inserted in the membrane where they form hemichannels. Cx are protein products of a related gene family, which in humans contains 21 members 7. The expression pattern of Cx is regulated according to the tissue and the developmental and metabolic state 8. In the lens, the isoforms Cx43, Cx46, and Cx50 are expressed. Cx43 forms gap junction channels in the epithelial cell monolayer that covers the anterior surface of the lens. In the lens bulk, the gap junctions between the lens fibers are formed by Cx46 and Cx50 9. These gap junction channels are involved in the lens internal circulation system that is essential for the homeostasis of this avascular tissue 10. Mutations that change the function of Cx46 and Cx50 are associated with cataracts (summarized in 9), which stress the importance of gap junction channels in the lens. However, the functional link between mutation of the Cx and the phenotypic consequence is not always understood.

The mutation found at position 188 in the second extracellular loop (E2) domain of Cx46 has been linked to an autosomal dominant zonular pulverulent cataract 11. As dominantly inherited, it is possible that the mutant variant affects the co‐expressed wild‐type (wt) Cx and/or its interaction with other cellular components 9. Recent results showed that the Cx46N188T could oligomerize in connexons, which were trafficked to and inserted in the membrane in similar manner as connexons formed by the wt variant. However, it was found that the mutant was not able to form gap junction plaques, suggesting that the docking was affected. Structural modeling and molecular dynamics (MD) simulations of a human Cx46 (hCx46) model, using the crystallized Cx26 12 as template, indicate that N188 in a protomer within a connexon of one cell forms hydrogen bonds (HB) with residues R180, T189, and D191 of the counterpart protomer within the connexon in the adjacent cell to stabilize the docked hCx46 gap junction channel. Mutation of the asparagine to T188 led to a decrease in the number of HBs and destabilized the favorable interaction between the connexons 13. In multimeric protein complex, mutated subunits might oligomerize with wts and cause thereby the mutated phenotype 14. The mutant might not affect the trafficking to and the insertion in the membrane. We proposed to use concatenated hCx46wt‐hCx46N188T and hCx46N188T‐hCx46wt to analyze how hCx46N188T affected co‐expressed hCx46wt to achieve a dominant inheritance. Concatenation was shown to be a good technique that allows to gain insight in the architecture of multimeric membrane proteins such as acetyl choline or γ‐aminobutyric acid receptors as well as K^+^ channels or Cx 15, 16, 17, 18, 19, 20, 21.

In combination with MD simulation, the results of concatenated heterodimers (hCx46wt‐hCx46N188T and hCx46N188T‐hCx46wt) suggest that the presence of the hCx46N188T in hemichannels reduces the HBs between the hemichannels of adjacent lens fibers, thereby lowering the number of gap junction channels between the cells.

## Materials and methods

### Molecular biology

For the transfection of the HeLa cells, the destination plasmid pEF‐I‐GFP GX, which allowed the co‐expression of untagged Cx together with a GFP, and the psDEST47 were used. pEF‐I‐GFP GX 22 was a gift from John Brigande (Addgene plasmid # 45443, Watertown, MA, USA). psDEST47 was created by using a ‘reverse’ BP‐cloning reaction with the expression clone pcDNA‐DEST47‐GFP‐GFP and the pDONR221 linearized with EcoNI. pcDNA‐DEST47‐GFP‐GFP 23 was a gift from Patrick Van Oostveldt (Addgene plasmid # 36139). The psDEST47 was transformed into *Escherichia coli* BD3.1 cells and selected on ampicillin and chloramphenicol containing LB‐Agar plates. The purified psDEST47 was used to create a C‐terminally GFP‐labeled fusion protein via the LR‐cloning reaction. For the molecular cloning, the multisite Gateway Pro kit was used (Thermo Fisher Scientific, Waltham, MA, USA). To generate the various Entry plasmids for the gateway cloning, the hCx46 and hCx46N188T 13 genes were used as template for the PCR (Phusion; Thermo Fisher Scientific) with the primers listed in Table 1 followed by the BP‐Clonase reaction (Thermo Fisher Scientific). The attB2 R stop primer was used for the pEF‐I‐GFP GX plasmids. The ten Entry plasmids were transformed into *E. coli* MachI cells. The twelve expression clones were generated by LR‐cloning (LR Clonase II plus; Thermo Fisher Scientific) with the purified Entry vectors and the aforementioned destination plasmids followed by a transformation into *E. coli* MachI cells. All gateway reactions were performed in a total volume of 2.5 μL. The cloning was verified by sequencing (Seqlab, Göttingen, Germany).

**Table 1:** Primers used for the PCR to generate the entry clones by BP‐cloning

Primer	5′–3′ sequence
GW_BP‐cloning hCx46 attB1 F	GGGGACAAGTTTGTACAAAAAAGCAGGCTCCATGGGCGACTGGAGCTTTCTGG
GW_BP‐cloning hCx46 attB2 R	GGGGACCACTTTGTACAAGAAAGCTGGGTGGGCCCGCGGTACCGTCGAC
GW_BP‐cl. hCx46 stop attB2 R	GGGGACCACTTTGTACAAGAAAGCTGGGTTCTAGATGGCCAAGTCCTCCGGT
GW_BP‐cloning hCx46 attB5r R	GGGGACAACTTTTGTATACAAAGTTGTGGCCCGCGGTACCGTCG
GW_BP‐cloning hCx46 attB5 F	GGGGACAACTTTGTATACAAAAGTTGTAATGGGCGACTGGAGCTTTCTGG

### Cell culture

For the cultivation of HeLa cells (DSMZ no.: ACC 57, Leibniz Institute DSMZ—German Collection of Microorganisms and Cell Cultures, Braunschweig, Germany), Dulbecco's modified Eagle's medium/Ham's F12 (1 : 1) medium (FG 4815; Biochrom, Berlin, Germany) supplemented with 10% fetal bovine serum (Biochrom), 1 mg·mL^−1^ penicillin, and 0.1 mg·mL^−1^ streptomycin (Biochrom) was used. HeLa cells were cultured in a humidified atmosphere with 5% CO_2_ at 37 °C. Every 2–3 days, the medium was renewed.

### Transfection

For the transfection of one well of a 24‐well plate, 500 ng plasmid and 1.5 μL FuGene HD (Promega, Mannheim, Germany) were incubated in 25 μL OptiMEM I medium (Thermo Fisher Scientific) for 15 min at RT and added to the HeLa cells, which were washed with 500 μL OptiMEM I medium prior transfection. After 4–6 h, the medium was exchanged to the antibiotic‐free culture medium.

### Quantification of the gap junction plaques

A day prior to imaging, 7 × 10^4^ HeLa cells were grown on collagen‐I‐coated coverslips and transfected with the psDEST47 plasmids. The cells were fixed with 3.7 % formaldehyde and stained with Hoechst 33342 (1 μg·mL^−1^; Sigma‐Aldrich, St. Louis, MO, USA) and Alexa 555‐conjugated Wheat Germ Agglutinin (5 μg·mL^−1^; Molecular Probes, Eugene, OR, USA) to improve the visibility of the cell‐cell contact regions. For the imaging, a confocal Nikon Eclipse TE2000‐E C1 laser scanning microscope (Nikon GmbH, Düsseldorf, Germany) was used as described previously 13, 24, 25. For each variant, at least 50 cell pairs were analyzed from at least four different transfections. The number of gap junction plaques was evaluated by using fiji 26. The data are given as mean ± SEM and evaluated using Student's *t*‐test.

### Dye uptake

A day prior the dye uptake experiment, subconfluent HeLa cells grown on collagen‐I‐coated coverslips (10 mm) were transfected with the pEF‐I‐GFP plasmids. The GFP fluorescence was used to define the ROIs in which ethidium bromide (Etd) dye uptake was followed. A coverslip was placed in a chamber containing 400 μL bath solution composed of (in mm) 121 NaCl, 5.4 KCl, 25 HEPES, 0.8 MgCl_2_, 5.5 glucose, 6 NaHCO_3_, 2 CaCl_2_, pH 7.4. The chamber was mounted on an inverted fluorescence microscope Nikon Ti‐E (Nikon GmbH) equipped with a monochromator Polychrome V (TILL I.D. GmbH, Planegg, Germany) and a CCD camera Orca Flash 4.0 (Hamamatsu Photonics Deutschland GmbH, Herrsching, Germany). After determination of ROIs, the cells were perfused with a prewarmed (37 °C) bath solution containing 5 μm Etd, at a flow rate of 1 mL·min^−1^. Ten minutes after the beginning of the experiment, a Ca^2+^‐ and Mg^2+^‐free bath solution with Etd was applied for 10 min, followed by a Ca^2+^‐ and Mg^2+^‐free bath solution containing Etd and 1 mm La^3+^ for further 10 min. Fluorescence images were taken every 15 s during the whole experiment (30 min). The rate of Etd uptake for each experimental section was estimated by evaluating the change in fluorescence intensity in the cells between 4–9 min, 14–19 min, and 24–29 min, respectively. The results are given as mean values ± SEM. The significance of the difference was evaluated by an ANOVA and a post hoc Tukey test (*** for *P* ≤ 0.001 and * for *P* ≤ 0.05).

### Dye transfer experiments

Dye transfer experiments were performed with Lucifer yellow (LY, 1 mg·mL^−1^) using the whole‐cell patch‐clamp technique as previously described 24, 25. The dye coupling is given as average ratio of the sum of tested pairs (*n*) for at least four transfections for each variant. To evaluate the significance of the difference, Student's *t*‐test was used.

### Structural modeling and molecular dynamics simulations

All‐atom structural models of hCx46 gap junction channels were generated as described earlier 13. The amino acid asparagine 188 was mutated to threonine without disturbing the backbone geometry, and the structures were prepared and optimized using the Protein Preparation Wizard and Macromodel of the Schrödinger Suite 2018‐1 (Schrödinger Release 2018‐1: Maestro, Protein Preparation Wizard, Epik, Macromodel, Schrödinger, LLC, New York, NY, USA). Mixed gap junction channels of wt and N188T mutated Cx were prepared in two arrangements, channel 1 with alternating hCx46wt and hCx46N188T Cx per hemichannel and differing types of Cx binding each other at the connexon interface, while channel 2 featured identical types of Cx of the hemichannels facing each other at the binding interface. The TIP3P water model 27 was used to immerse the gap junction channels in a rectangular water box, extending up to 10 Å from the proteins, and Cl^−^ anion was added to keep the net system charge neutral. Each system comprised a total of ~ 210 000 atoms. MD simulations were performed with NAMD2.12 28 and the CHARMM36 all‐atom additive force field 29. Simulations were conducted in a NpT ensemble with a constant temperature of 310 K and pressure at 1 atm using Langevin dynamics and the Langevin piston method. The RATTLE algorithm was used to constrain all covalent bonds. Velocity Verlet integration was used with a time step of 2 fs. Long‐range electrostatics was treated using the particle‐mesh Ewald method 30 and a short‐range cutoff of 12 Å was used for nonbonded interactions. The solvated and neutralized gap junction channel systems were initially energy minimized and subsequently equilibrated at 310 K and 1 atm. After reaching a converged root mean square deviation of the protein backbone atoms, 100‐ns MD production runs for each channel were carried out.

## Results

An adenine–cytosine (A → C) replacement at position 563 in the coding sequence of hCx46 causes an exchange of amino acid residue asparagine to threonine at position 188 (N188T). This mutation is associated with an autosomal dominant congenital nuclear pulverulent cataract 11. We transfected hCx46wt and hCx46N188T as well as concatenated hCx46wt‐hCx46wt, hCx46N188T‐hCx46N188T, hCx46wt‐hCx46N188T, and hCx46N188T‐hCx46wt in HeLa cells and analyzed the functional consequences of the mutation on the gap junction hemichannels and cell‐cell coupling gap junction channels that may contain both Cx isoforms in combination with MD simulations.

**Figure 1:**
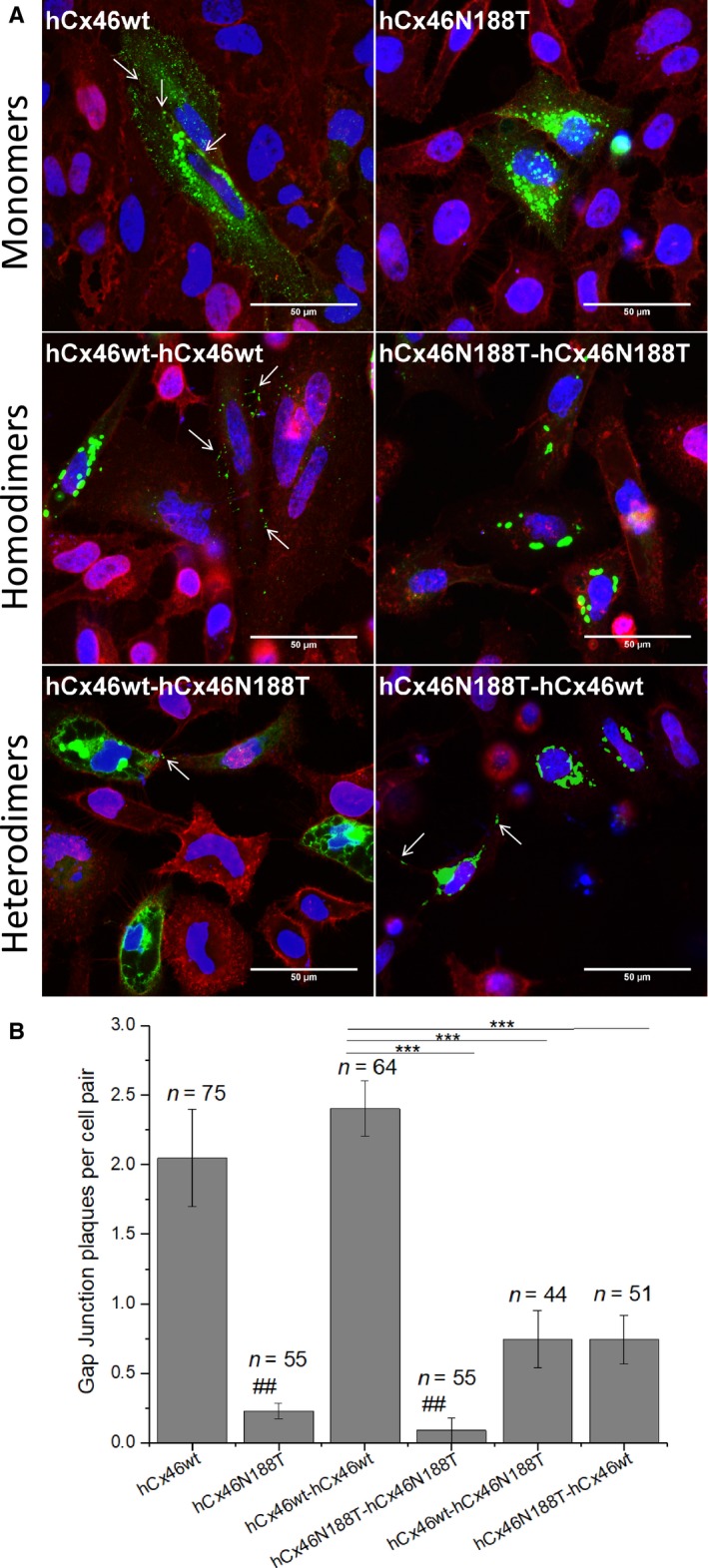
Formation of gap junction plaques by the different variants. (A) Representative micrographs of the HeLa cells expressing eGFP‐labeled hCx46wt, hCx46N188T, and the four possible homodimeric and heterodimeric tandems. The cells were stained with Hoechst 33342 (nuclei; blue) and WGA‐Alexa‐Fluor® 555 (Molecular Probes) (membrane; red). Gap junction plaques are indicated by arrows. The cell indicated by an asterisk (bottom left panel) shows a green GFP label distributed all over the cell membrane. Such single cells were occasionally found for all variants. They probably represent excessive overexpression of the transfected protein. Scale bar = 50 μm. (B) Quantification of the number of gap junction plaques formed by eGFP‐labeled hCx46 monomers and the four different homo‐ and heterodimers between HeLa cell pairs. imagej (U. S. National Institutes of Health, Bethesda, MD, USA) was used for the analysis. The average number of gap junction plaques per cell pair for the different variants is given as for *n* considered pairs in at least three transfection experiments for each variant. Error bars represent the SEM. The significance of difference between the variants and hCx46 (^##^ *P* ≤ 0.01) or between the variants and hCx46wt‐hCx46wt (***P ≤ 0.001) was evaluated by Student's *t*‐test.

**Figure 2:**
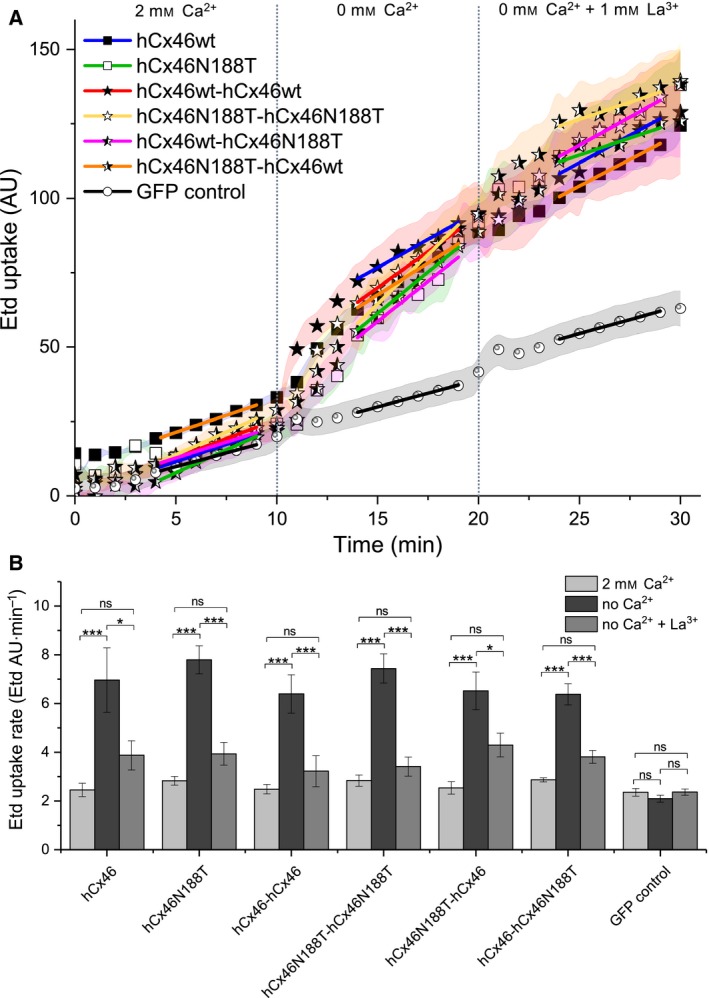
Analysis of the functionality of the hemichannels by dye uptake experiments. HeLa cells expressing the IRES‐GFP Plasmids grown on coverslips to a confluency of about 40–50% were used to perform dye uptake experiments with 5 μm Etd. (A) The cells were perfused with media containing 2 mm Ca^2+^, no Ca^2+^, or no Ca^2+^ but 1 mm La^3+^ for 10 min each. The mean fluorescence signal of Etd during the dye uptake experiments showed that all tested hemichannels allowed dye uptake when Ca^2+^ was removed. The data are given as average for at least ten experiments for each construct in at least three different transfections for each construct, resulting in at least 190 analyzed cells. The error bars represent the SEM. (B) The dye uptake rate was quantified for all tested constructs and the backbone control in presence or absence of Ca^2+^ and La^3+^. For each variant, at least 190 cells from at least three independent transfections were analyzed. The data were evaluated by using a one‐way ANOVA followed by a Tukey test (*P ≤ 0.05, ***P ≤ 0.001). The error bars represent the SEM.

For the analysis of the gap junction plaque number, the GFP‐labeled variants were expressed in HeLa cells. Although the transfection efficiency of about 20–30% for all observed variants did not differ, the number of the formed gap junction plaques differed strongly (Fig. 1). Cells expressing the monomer hCx46wt and the homodimer hCx46wt‐hCx46wt formed the most plaques (Fig. 1). By counting, we found an average number of about two gap junction plaques per cell pair expressing hCx46 monomer. A slight (not significant) increase to about 2.4 gap junction plaques per cell pair was observed for cells expressing the homodimer hCx46wt‐hCx46wt (Fig. 1). Although the individual area of the plaques formed by the concatenated Cx was reduced, the number of gap junction plaques was not affected by the concatenation. In our previous study (21), we showed that the concatenated Cx are able to do the trafficking of hemichannels to the membrane and the formation of functional channels between cells. On the other side, in agreement with previous results (13), here we found that hCx46N188T rarely formed gap junction plaques (Fig. 1). Likewise, gap junction plaques between cells expressing the homodimer hCx46N188T‐hCx46N188T were extremely rare (Fig. 1). A quantification showed an average of about 0.2 gap junction plaques between two adjacent cell expressing the hCx46N188T monomers. For cells expressing the homodimeric hCx46N188T, an average of about 0.1 gap junction plaques was counted (Fig. 1). For the heterodimers, the probability to form gap junction plaques was strongly increased in comparison with the homodimer hCx46N188T‐hCx46N188T but clearly decreased compared to the hCx46wt‐hCx46wt homodimer. It is possible that the presence of the hCx46N188T either as homomer or as tandem in any combination strongly reduced the presence of the protein in the membrane. As for the hCx46N188T homomer, previous results showed that when expressed in *Xenopus* oocytes or HeLa cells, hCx46N188T caused a voltage dependent current comparable in amplitude with the current caused by the hCx46wt 13. Moreover, by analyzing the dye uptake capacity of the cells expressing the monomers composed of hCx46wt and hCx46N188T, or the homodimers hCx46wt‐hCx46wt and hCx46N188T‐hCx46N188T or the heterodimers hCx46wt‐hCx46N188T and hCx46N188T‐hCx46wt, we found a similar dye uptake rate before and after reducing external Ca^2+^ in cells expressing either variant (Fig. 2).

**Figure 3:**
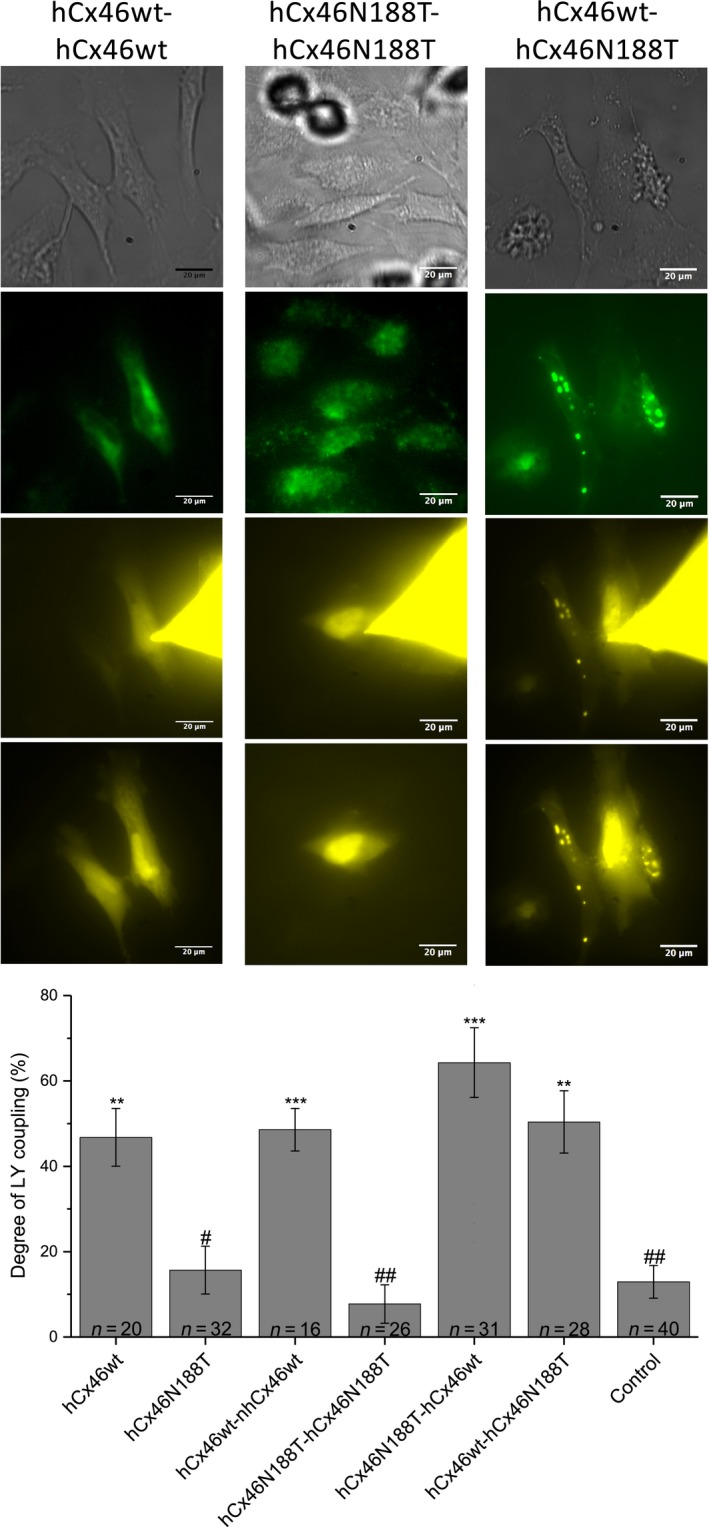
Dye transfer experiments to analyze the gap junction functionality. A whole‐cell patch‐clamp configuration with pipette filling solution containing LY (1 mg·mL^−1^) was established on one cell of a cell pair expressing the different variants. Mock transfected HeLa cells were used as control. Representative micrographs of cells expressing hCx46wt‐hCx46wt, hCx46N188T‐hCx46N188T, or hCx46wt‐hCx46N188T before (first and second row, phase contrast, and GFP, respectively), during (third row, LY signal 5 min after establishment of the whole‐cell configuration) and after (fourth row, 10 min after removal of the dye filled capillary) dye coupling experiments are shown. Scale bar = 20 μm. The cells were considered as coupled if the fluorescence intensity, which was measured in the unpatched cell of a cell pair after 10 min, was at least twice as bright as the background which was measured at the beginning of the experiment. The probability of dye coupling (bar plot) was estimated as a ratio of the sum of LY coupled cell pairs and the total amount of tested cell pairs. The average percentages of the LY dye coupling for the different variants for *n* considered cell pairs in at least three transfection experiments for each variant is given. The error bars represent the SEM. The significance of the difference to control cell pairs which did not express any variant (**P ≤ 0.01, ***P ≤ 0.001) and to cells pairs expressing hCx46wt (^#^ *P* ≤ 0.05, ^##^ *P* ≤ 0.01) was evaluated by Student's *t*‐test.

At the cell‐to‐cell gap junction level, the dye transfer experiments showed that the gap junction plaques whether they were formed by hCx46wt, hCx46wt‐hCx46wt homodimers, hCx46wt‐hCx46N188T, or hCx46N188T‐hCx46wt contained gap junction channels that allowed the transfer of LY from one cell to the adjacent cell (Fig. 3). LY transfer was observed in about 50% of the cell pairs. In contrast, in cells expressing the monomeric hCx46N188T and the homodimeric hCx46N188T‐hCx46N188T, the probability of dye transfer did not significantly exceed that of control cells, which were MOCK transfected. Dye transfer was observed in about 10% of these cell pairs (Fig 3). The 10% of dye coupling in MOCK transfected cells is mostly related to a background which is not affected by gap junction inhibitors such as carbenoxolone.

**Figure 4:**
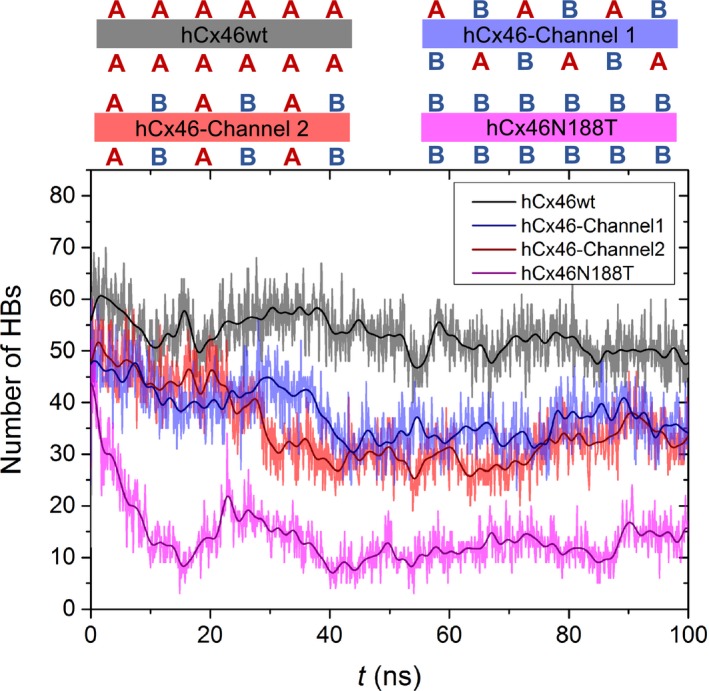
Number of HBs between interacting connexons over simulation time. Composition of the docking interfaces of the four gap junction channels are shown at the top with a red capital A representing a hCx46wt Cx and the blue capital B depicting hCx46N188T Cx. An average of 53 HBs were found to stabilize the hCx46wt connexon binding interface (black) over a total simulation time of 100‐ns MD simulations, while 31 HBs were found for the hCx46wt‐hCx46N188T interacting with hCx46wt‐hCx46N188T (red) and 35 HBs with hCx46N188T‐hCx46wt (blue) connexons, respectively. The number of HBs for hCx46N188T connexons interacting with other hCx46N188T connexons (purple) decreased to an average of 12 HBs within the 100 ns.

Previously, we analyzed the stability of the interaction in a structural model of docked hCx46 Cx, derived from the crystal structure of hCx26, *in silico* 12. We could show that the hCx46N188T mutation destabilized the Cx interaction, which indicated that the docking of mutated connexons of adjacent cells might be affected 31. In the present report, we extended our study by classical MD simulations, including hexamers composed of either hCx46wt, hCx46N188T, or alternating hCx46wt and hCx46N188T protomers. Monitoring the number of interactions along the MD trajectories showed an average of 53 HBs of the hCx46wt connexons interacting with a second hCx46wt connexon. The overall number of stabilizing HBs between hCx46N188T hexamers markedly decreased in the first 40 ns of the simulations and reached a plateau around an average of 12 HBs over the 100‐ns simulation time (Fig 4). For both gap junction channels with hCx46wt and hCx46N188T building the connexons, a reduction in the number of HBs was observed as compared to hCx46wt, which fluctuated around 31–35 HBs between the docked connexons (Fig. 4). These results suggested that the presence of the hCx46N188T would reduce the efficiency to form hCx46 gap junction channels between lens fibers.

## Discussion

The N188T mutation is located in the E2 domain of human lens Cx46 and has been linked to an autosomal dominant zonular pulverulent cataract 11. As dominantly inherited disease, it is possible that the mutant variant affects the co‐expressed wt Cx and/or its interaction with other cellular components 9. Our previous results showed that the hCx46N188T mutant did not affect hexamerization in connexons and the trafficking to and insertion of the connexons in the membrane to form gap junction hemichannels. Electrophysiological experiments showed that hCx46wt and hCx46N188T expressed in *Xenopus* oocytes or HeLa cells formed hemichannels, which responded to depolarizing voltages by similar currents 13. The present report supports the previous data using dye uptake experiments. As shown, similar dye uptake rates were observed in cells expressing hCx46N188T as compared to cells expressing hCx46wt (Fig. 2). The results indicate that an effect of the hCx46N188T mutation on association of the Cx with other proteins such as those involved in trafficking is unlikely. By observing the formation of gap junction plaques formed by GFP‐labeled hCx46wt and hCx46N188T, we found that hCx46N188T hemichannels had a problem with the docking of the channels 13; therefore, we hypothesized that the co‐expression of the wt and the mutant variant would negatively affect the docking of the hemichannels. For the heterodimeric concatenated hCx46wt‐hCx46N188T and hCx46N188T‐hCx46wt, a reduced number of gap junction plaques compared to the homodimeric concatenated hCx46wt‐hCx46wt variant could be observed, even if the heterodimeric concatenated variants showed an increased number of gap junction plaques compared to the homodimeric concatenated hCx46N188T‐hCx46N188T (Fig. 1). Concerning the number of gap junction plaques, the concatenation as technique did not alter the number of gap junction plaques, since we observed that the concatenated homodimers hCx46wt‐hCx46wt and hCx46N188T‐hCx46N188T showed a similar number of gap junction plaques like the hCx46wt and hCx46N188T monomers, respectively (Fig. 1).

**Figure 5:**
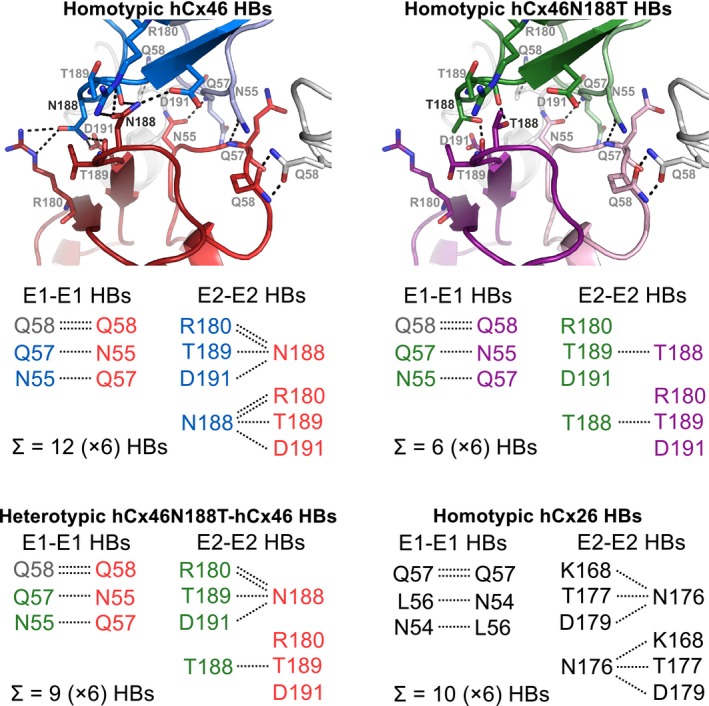
HB network of the Cx–Cx interaction. The HB network of Cx26 (black, lower right side) is adopted from 12, 32. For a better comparison of the relevant amino acid residues, all homologue residues are displayed. The nondynamic hydrogen bound network of the docking of hCx46 (red and blue) shows 12 relevant HBs per Cx–Cx interaction. Eight HBs were formed in the E2–E2 interaction and four were built for the E1–E1 interaction. Note, that all of the E2‐HBs are formed by the residue N188. A channel of hCx46 is theoretically stabilized by 72 HBs. When the hCx46 is mutated at position N188T (green and purple), only 36 HBs could theoretically stabilize the docked channels. The heterotypic hCx46wt‐hCx46N188T (green and red) connexon is theoretically stabilized by 54 HBs.

The structural models have showed that the docking between connexons required a tight interaction network, mostly governed by HB interactions. The crystallized Cx26 showed that the N176 residue in the E2 domain formed HBs with K168, T177, and D179 of the opposing Cx molecule in the counterpart connexon of the adjacent cells 12, 32. According to the model, 6 HBs were formed between the E2 domains of the interacting Cx26 protomers between the docked hemichannels. Moreover, further 4 HBs were found in the first extracellular loop (E1) domains, where a protomer interacted with two Cx, resulting in a total of 10 HBs per Cx–Cx interaction or 60 HBs per connexon–connexon interaction 32, 33, 34, 35, 36, 37. For hCx46, N188 is homologous to N176 of Cx26. As shown in Fig. 5, the model predicted that the residue N188 of the E2 loop in a hCx46 molecule in a connexon of one cell forms HBs with residues R180 (two HBs), T189 (one HB), and D191 (one HB) of the opposing Cx molecule in the counterpart connexon of the adjacent cells 13, 31. As for the Cx26, 4 HBs would be formed between the E1 domains of a Cx46 (Fig. 5). As results, a maximum of 72 HBs per connexon–connexon interaction can be assumed for one hCx46wt channel. MD modeling showed that the sum of HBs oscillated between 42 and 68 between the adjacent connexons formed by hCx46wt (Fig. 4, black trace). The formed gap junction channels are stabilized enough to allow a dye coupling (Fig. 3) and an assembly in the gap junction plaques (Fig. 1). In contrast, the MD simulation showed that the sum of HBs between adjacent connexons composed of hCx46N188T oscillated between 24 and only four HBs (Fig. 4, pink trace). These HBs, which might be mostly contributed by interactions in E1 domains, do not support stable gap junction channels and corollary gap junction plaques cannot be formed (Fig. 1). In case of hCx46wt and hCx46N188T co‐expression, depending on the stoichiometry, different scenarios can be envisaged. For a simplistic model, we considered interactions between hemichannels formed by alternating hCx46wt and hCx46N188T, which also reflects the situation of our experiments with the concatenated heterodimers. Two alternatives are assumed for the formed gap junction channels: wt–mutant interaction (Fig. 4, blue trace) or wt–wt and mutant–mutant (Fig. 4, red trace) interactions. For both alternatives, the MD modeling found that the sum of HBs oscillated between 25 and 45 between the adjacent connexons.

Using a combination of MD modeling and cellular experiments, it was shown that manipulations that reduced the possibility of the formation of HBs between the E2 regions of Cx26 protomers by more than two HBs per Cx–Cx interaction were not functional 35. How this HB reduction in E2 would affect the total HB binding network between protomers and connexons is so far not clear. However, the results suggest that a minimum number of HBs must be required for stable connexon docking. In this scenario, the mixed hCx46wt/hCx46N188T channels might be affected by this threshold with the consequence of a reduction of the probability to form stable gap junction channels, which correlates with reduction of the number of gap junction plaques as shown in Fig. 1. However, even these reduced gap junction plaques contained gap junction channels that allowed dye transfer experiments (Fig. 3). The dye transfer experiments are evaluated as all‐or‐nothing process, which shows only that there are gap junction channels between two cells but says almost nothing concerning the density of the gap junction channels. Since we consider cell pairs expressing the variants, the experiments show therefore that concatemers containing N188T together with the wt support LY transfer as well as the wt. Moreover, the observation that the hetero‐concatemers hCx46wt‐hCx46N188T formed channels that allowed dye coupling agrees very well with the observation that hCx46wt and hCx46N188T formed gap junction hemichannels with similar properties (Fig. 2 and 13). Therefore, we postulate that cells expressing only hCx46N188T will not form gap junction channels and cells that might co‐express hCx46wt and hCx46N188T will form fewer gap junction channels than cells expressing only the wt variant.

## Conclusion

The hCx46N188T mutant is dominantly inherited. The usage of concatenated hCx46wt and hCx46N188T in combination with MD simulations shows that the mutation N188T might be negative dominant 11, since the mutant variant might oligomerize with the wt in hemichannels that would inefficiently dock to each other to form gap junction channels between lens fibers. Cx46 gap junction channels participate in lens internal circulation system that is essential for the metabolic homeostasis and, thus, the physiology of the avascular lens 10. By reducing the formation of hCx46 channels between lens fiber cells, the hCx46N188T mutant would therefore favor the development of a cataract. The degree of participation of the N188T isoform to gap junction channels *in vivo* situation is not known at yet. However, the finding that N188T mutation was linked to a pulverulent form of cataract 11 might reflect an uneven participation of the mutant isoform in the formation of gap junction channels.

## Conflict of interest

The authors declare no conflict of interest.

## Author contributions

PS and AN conceptualized the study; PS performed the data curation, and formal analysis; AN performed the funding acquisition and project administration; PS and YS performed the investigation of the article; PS, MP, and AN performed the methodology; MP performed the MD simulations; PS and MP performed the visualization; PS, MP and AN wrote the original draft of the manuscript; PS and AN wrote, reviewed, and edited the article. The paper was proofread by all authors.
